# LGI1 Autoantibodies Enhance Synaptic Transmission by Presynaptic K_v_1 Loss and Increased Action Potential Broadening

**DOI:** 10.1212/NXI.0000000000200284

**Published:** 2024-08-14

**Authors:** Andreas Ritzau-Jost, Felix Gsell, Josefine Sell, Stefan Sachs, Jacqueline Montanaro, Toni Kirmann, Sebastian Maaß, Sarosh R. Irani, Christian Werner, Christian Geis, Markus Sauer, Ryuichi Shigemoto, Stefan Hallermann

**Affiliations:** From the Carl-Ludwig-Institute of Physiology (A.R.-J., F.G., T.K., S.M., S.H.), Faculty of Medicine, Leipzig University; Section Translational Neuroimmunology (J.S., C.G.), Department of Neurology, Jena University Hospital; Department of Biotechnology and Biophysics (S.S., C.W., M.S.), University of Würzburg, Biocenter, Germany; Institute of Science and Technology Austria (ISTA) (J.M., R.S.), Klosterneuburg, Austria; Oxford Autoimmune Neurology Group (S.R.I.), Nuffield Department of Clinical Neurosciences, University of Oxford, ; Department of Neurology (S.R.I.), John Radcliffe Hospital, Oxford University Hospitals, United Kingdom; and Departments of Neurology and Neurosciences (S.R.I.), Mayo Clinic Jacksonville, FL.

## Abstract

**Background and Objectives:**

Autoantibodies against the protein leucine-rich glioma inactivated 1 (LGI1) cause the most common subtype of autoimmune encephalitis with predominant involvement of the limbic system, associated with seizures and memory deficits. LGI1 and its receptor ADAM22 are part of a transsynaptic protein complex that includes several proteins involved in presynaptic neurotransmitter release and postsynaptic glutamate sensing. Autoantibodies against LGI1 increase excitatory synaptic strength, but studies that genetically disrupt the LGI1-ADAM22 complex report a reduction in postsynaptic glutamate receptor-mediated responses. Thus, the mechanisms underlying the increased synaptic strength induced by LGI1 autoantibodies remain elusive, and the contributions of presynaptic molecules to the LGI1-transsynaptic complex remain unclear. We therefore investigated the presynaptic mechanisms that mediate autoantibody-induced synaptic strengthening.

**Methods:**

We studied the effects of patient-derived purified polyclonal LGI1 autoantibodies on synaptic structure and function by combining direct patch-clamp recordings from presynaptic boutons and somata of hippocampal neurons with super-resolution light and electron microscopy of hippocampal cultures and brain slices. We also identified the protein domain mediating the presynaptic effect using domain-specific patient-derived monoclonal antibodies.

**Results:**

LGI1 autoantibodies dose-dependently increased short-term depression during high-frequency transmission, consistent with increased release probability. The increased neurotransmission was not related to presynaptic calcium channels because presynaptic Ca_v_2.1 channel density, calcium current amplitude, and calcium channel gating were unaffected by LGI1 autoantibodies. By contrast, application of LGI1 autoantibodies homogeneously reduced K_v_1.1 and K_v_1.2 channel density on the surface of presynaptic boutons. Direct presynaptic patch-clamp recordings revealed that LGI1 autoantibodies cause a pronounced broadening of the presynaptic action potential. Domain-specific effects of LGI1 autoantibodies were analyzed at the neuronal soma. Somatic action potential broadening was induced by polyclonal LGI1 autoantibodies and patient-derived monoclonal autoantibodies targeting the epitempin domain, but not the leucin-rich repeat domain.

**Discussion:**

Our results indicate that LGI1 autoantibodies reduce the density of both K_v_1.1 and K_v_1.2 on presynaptic boutons, without actions on calcium channel density or function, thereby broadening the presynaptic action potential and increasing neurotransmitter release. This study provides a molecular explanation for the neuronal hyperactivity observed in patients with LGI1 autoantibodies.

## Introduction

Autoimmune encephalitis is a growing group of diseases caused by autoantibodies against various neuronal antigens, collectively leading to severe mental and behavioral disorders.^1,2^ Autoimmune encephalitis with a predominant phenotype of limbic system involvement (so-called limbic encephalitis) primarily affects the mesial temporal lobe, hippocampus, and amygdala and is characterized by focal and generalized seizures and limbic dysfunction including mood changes and amnesia. The most frequent type of limbic encephalitis is caused by autoantibodies against the neuronal protein leucine-rich glioma inactivated 1 (LGI1)^2^ resulting in characteristic faciobrachial dystonic and generalized seizures together with amnestic deficits. Seizures rapidly respond to immunotherapy, while patients often develop progressive cognitive impairment and hippocampal sclerosis if treatment is delayed.^3-6^ Besides its major role in limbic encephalitis, genetic variations in LGI1 have been linked to an inherited form of epilepsy which involves the lateral temporal lobe.^7-9^

LGI1 has 2 main domains, a N-terminal leucine-rich repeat (LRR) and a C-terminal epitempin (EPTP) domain.^10^ The EPTP domain interacts with presynaptic and postsynaptic ADAM22-family receptors.^11-13^ LGI1-ADAM22 heterodimers have been suggested to dimerize in the synaptic cleft through an LRR-EPTP interaction, thus linking LGI1-ADAM22s within presynaptic and postsynaptic membranes to form a transsynaptic-tetrameric complex.^12^ ADAM22 receptors have been reported to interact directly or indirectly with both, presynaptic proteins including CASK, SAP97, and various pore-forming or accessory K_v_1 and Ca_v_ channel subunits, and the postsynaptic neurotransmitter receptor scaffold including PSD95 and glutamate receptors.^14,15^ In addition, LGI1 was found to be critical for potassium channel expression^16^ and function.^17^ The transsynaptic LGI1-ADAM22 complex was therefore proposed as a key component controlling presynaptic transmitter release to postsynaptic receptors.^18^

To study the function of LGI1 at synapses and the consequences of disturbed LGI1 signaling, 2 main approaches have been adopted. First, genetically modified cell lines or animal models were used either that overexpressed LGI1,^19^ did not express LGI1,^11,20,21^ or that harbored genetic variations of LGI1 associated with inherited epilepsy.^11,19,22,23^ In most of these studies, LGI1 overexpression enhanced and LGI1 loss reduced the postsynaptic α-amino-3-hydroxy-5-methyl-4-isoxazolepropionic acid (AMPA) receptor response and receptor clustering.^11,13,22,24^ Furthermore, LGI1 loss increased the presynaptic neurotransmitter release.^16,19,21^ Second, synaptic LGI1 function was studied using patient-derived polyclonal LGI1 autoantibodies^25-28^ or domain-specific monoclonal autoantibodies.^29,30^ Reminiscent of genetically induced LGI1 loss, treatment with autoantibodies reduced postsynaptic AMPA receptors in primary hippocampal cultures^26^ and acute hippocampal brain slices.^27^ Furthermore, recent evidence indicates that LGI1 autoantibodies increase presynaptic release probability and overall synaptic strength^27-29^ with strengthening paralleled by potassium channel loss.^27,30^ However, the mechanism by which LGI1 autoantibodies strengthen presynaptic neurotransmitter release and the responsible molecular domains remain elusive.

Here, we combined electrophysiologic somatic and subcellular presynaptic recordings from cultured hippocampal neurons with stimulated emission depletion (STED) microscopy,^31^ expansion microscopy (ExM) together with structured illumination microscopy (SIM),^32,33^ and electron microscopy to identify mechanisms involved in the LGI1 autoantibody-mediated increase in presynaptic release. We find that polyclonal LGI1 autoantibodies increase presynaptic release probability independent of calcium channels. By contrast, polyclonal LGI1 autoantibodies reduce presynaptic K_v_1.1 and K_v_1.2 channels and lead to increased action potential broadening, an effect replicated by monoclonal EPTP, but not LRR autoantibodies.

## Methods

### Standard Protocol Approvals, Registrations, and Patient Consents

Animal experiments were performed in accordance with the ARRIVE guidelines, and animals were handled according to the regulations of the Federal Saxonian (license # T29/19), Thuringian (licence # UKJ-17-053), and Bavarian state authorities (license # 55.2.2-2532-2-811) and in accordance with European regulations (Directive 2010/63/EU). All patients provided informed consent for use of plasma exchange material, and use of human material was approved by the local ethics committee of Jena University Hospital (licence # 2019-1415-Material).

### Data Availability

Data that support the findings of this study are available from the corresponding author on reasonable request.

## Results

### LGI1 Autoantibodies Induce a Dose-Dependent Increase in Synaptic Release Probability

**Figure 1:**
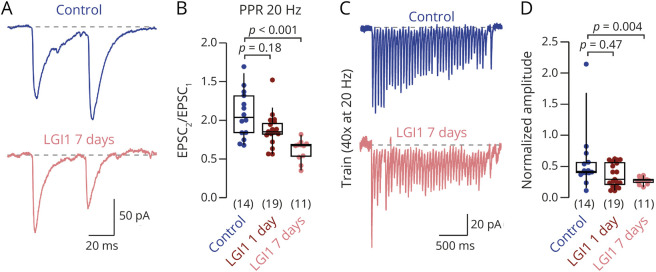
LGI1 Autoantibodies Induce a Dose-Dependent Increase in Synaptic Release Probability (A) Example paired EPSCs evoked at 20 Hz under control condition (blue) and following 7 days of LGI1 autoantibody treatment (rose). (B) Paired pulse ratio (PPR; amplitude of EPSC_2_/EPSC_1_) at 20 Hz under control condition (blue) and following 1 day or 7 days of LGI1 autoantibody treatment (red and rose, respectively). A nonparametric ANOVA (Kruskal-Wallis) test revealed *p* < 0.001. (C) Example EPSC trains (40 EPSCs evoked at 20 Hz) under control condition (blue) and following 7 days of LGI1 autoantibody treatment (rose). (D) Depression of normalized EPSC amplitudes during the late phase of the train (average of the last 10 train EPSCs). A nonparametric ANOVA (Kruskal-Wallis) test revealed *p* = 0.035. Numbers in brackets reflect recordings from individual neurons. Box plots cover percentile 25–75 with median indicated, whiskers indicate percentiles 10–90. The *p* values of the nonparametric ANOVA (Kruskal-Wallis) tests are provided in the legends, and the *p* values of the nonparametric post hoc tests (Dwass-Steel-Critchlow-Fligner pairwise comparisons) are provided in the figures. EPSCs = excitatory postsynaptic currents.

We first investigated the effect of polyclonal LGI1 autoantibodies on excitatory transmission in primary dissociated hippocampal cultures. Polyclonal LGI1 autoantibodies were obtained from the serum of 3 patients with LGI1 encephalitis and high titer of LGI1 antibodies and used as a pooled IgG fraction.^27^ Cultures were incubated with patient-derived polyclonal serum LGI1 autoantibodies included in the growth medium for 7 days (LGI1-7d; with a second dose applied 1 day before recordings), with LGI1 autoantibodies for 1 day only (LGI1-1d), or with patient control antibodies without antineuronal reactivity. We then recorded pharmacologically isolated excitatory postsynaptic currents (EPSCs) in somatic whole-cell voltage-clamp recordings evoked by external stimulation. As a measure of presynaptic release probability, we determined the paired-pulse ratio (PPR), which is largely independent of postsynaptic strength, synapse number, and neuronal morphology.^34^ EPSCs in control autoantibody-incubated neurons showed facilitation at frequencies of 20 Hz, reflected in PPR >1. Treatment with LGI1 autoantibodies reduced PPRs in a dose-dependent manner, indicating that LGI1 autoantibodies increase the synaptic release probability (Figure 1, A and B; median [IQR] PPR at 20 Hz: 1.03 [0.84–1.24], 0.85 [0.82–0.96], and 0.67 [0.53–0.69], n = 14, 19, and 11 for control, LGI1-1d, and LGI1-7d, respectively; nonparametric Kruskal-Wallis ANOVA test *p* < 0.001 and post hoc test *p* < 0.001 for control and LGI1-7d). Similarly, LGI1 autoantibodies reduced PPRs at 50 Hz stimulation (eFigure 1). To study autoantibody-induced changes in synaptic transmission in more detail, we analyzed short-term plasticity during evoked EPSC trains (50 EPSCs at 20 Hz; Figure 1C). LGI1 autoantibody treatment suppressed facilitation and induced faster and stronger depression of excitatory currents, in line with higher presynaptic release probability on autoantibody treatment (Figure 1D and eFigure 1; median [IQR] amplitude of the 10 last train EPSCs normalized to the first train EPSC: 0.42 [0.40–0.56] and 0.28 [0.24–0.29], n = 14 and 11, for control and LGI1-7d, respectively, post hoc *p* = 0.004). These data indicate a dose-dependent increase in presynaptic release probability on treatment with LGI1 autoantibodies. The amplitude of the initial train EPSC was not significantly different between control and autoantibody-treated neurons (median [IQR] EPSC amplitude: 223 [143–260] pA and 159 [105–210] pA, n = 14 and 11 for control and LGI1-7d, respectively, *p* = 0.15; data not shown). However, this is not surprising because the total EPSC amplitude besides release probability also depends on, e.g., postsynaptic strength, synapse number, and neuronal morphology, which may change on LGI1 loss. Because 7-day antibody treatment more robustly affected synaptic transmission than 1-day treatment, we adopted the 7-day treatment for subsequent analyses.

### LGI1 Autoantibodies Have Little Effect on Presynaptic Ca_v_2.1 Calcium Channel Density

**Figure 2:**
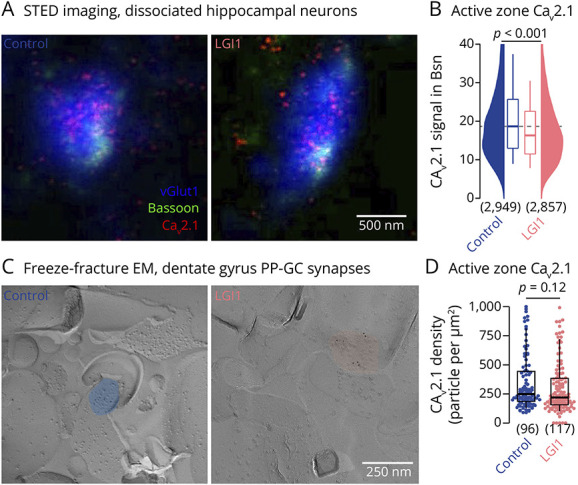
LGI1 Autoantibodies Have Little Effect on Presynaptic Ca_v_2.1 Calcium Channel Density (A) STED fluorescence images of presynapses stained for vGlut1 as a synaptic marker (blue), Bassoon as an active zone marker (green), and Ca_v_2.1 channels (red) treated with control antibodies (*left*) or with LGI1 autoantibodies (*right*). (B) Active zone Ca_v_2.1 fluorescence (within Bassoon) following control antibody (blue) and LGI1 autoantibody (rose) treatment. (C) Electron microscopic images of freeze-fracture replica immunolabeling for Ca_v_2.1 in hippocampal perforant path-granule cell (PP-GC) synapses for control antibody (*left*) and LGI1 autoantibody (*right*) treatment. (D) Active zone Ca_v_2.1 particle densities pooled from 3 animals per group (color code as in B). Numbers in brackets reflect individual active zones. Box plots cover percentile 25–75 with median indicated, whiskers indicate percentiles 10–90. *p* Values were calculated using the Mann-Whitney *U* test.

The release probability of presynaptic vesicles is influenced by the number, the position, and the properties of presynaptic calcium channels.^34-36^ Furthermore, proteome studies indicate interactions between the LGI1-receptor ADAM22 and calcium channels.^18,37^ Therefore, a straightforward explanation for the autoantibody-induced increase in release probability could be an increased presynaptic calcium influx due to either higher calcium channel abundance or faster channel gating. To first test whether LGI1 autoantibodies affected presynaptic calcium channel abundance, we performed STED imaging of Ca_v_2.1 channels, which is one of the main calcium channel types at hippocampal synapses.^38,39^ Ca_v_2.1 fluorescence signal intensities were quantified at excitatory presynapses (labeled by the vesicular glutamate transporter vGlut1, eFigure 2) and excitatory active zones (labeled by Bassoon within vGlut1-positive presynapses, Figure 2A). LGI1 autoantibodies decreased Ca_v_2.1 channel fluorescence intensities within both, presynapses and active zones (Figure 2B and eFigure 2; median [IQR] Ca_v_2.1 intensity within vGlut1-positive Bassoon: 18.7 [25.8–12.9] and 16.3 [22.6–11.4], n = 2949 and 2857 synapses for control and LGI1, respectively, *p* < 0.001). The ∼10% decrease in Ca_v_2.1 channel fluorescence intensity by LGI1 autoantibodies is in contrast to an increased synaptic release probability, which would require increased Ca_v_2.1 channel abundance instead.

To analyze the effect of LGI1 autoantibodies on Ca_v_2.1 channels within presynaptic active zones in more detail, we performed freeze-fracture replica immunoelectron microscopy of hippocampal presynapses from mice chronically infused with patient-derived polyclonal serum LGI1 autoantibodies using intraventricular osmotic pumps. LGI1 antibodies infused by osmotic pumps penetrated into the tissue and particularly into the hippocampus where they bound to their target antigen in contrast to a control antibody (eFigure 3). Antibody-treated mice did not develop obvious epileptic symptoms. We first analyzed dentate gyrus perforant path-granule cell synapses (Figure 2C) because LGI1 expression is highest in the dentate gyrus^17^ and transmission is affected presynaptically on genetic alteration of LGI1^19^ or LGI1 autoantibodies.^27^ Chronic LGI1 autoantibody infusion did not affect active zone Ca_v_2.1 channel density (Figure 2D; median [IQR] Ca_v_2.1 particle density per µm^2^: 248 [186–444] and 220 [156–386], 96 and 117 active zones for control and LGI1, respectively, from 3 animals each, *p* = 0.12) with a trend toward a ∼10% reduction on LGI1 autoantibody treatment, similar to STED recordings in cultured neurons. In addition, we quantified Ca_v_2.1 channel density at another LGI1-expressing synapse between dentate mossy fibers and CA3 neurons from chronically infused mice. Similarly, LGI1 autoantibodies did not affect Ca_v_2.1 channel density at these synapses (eFigure 2). These data show that LGI1 autoantibodies caused, if anything, a small reduction in Ca_v_2.1 channel density in boutons of hippocampal cultures and tissue. Therefore, alterations in calcium channels density cannot explain increased release probability by LGI1 autoantibodies.

### LGI1 Autoantibodies Do Not Affect Presynaptic Calcium Channel Gating

**Figure 3:**
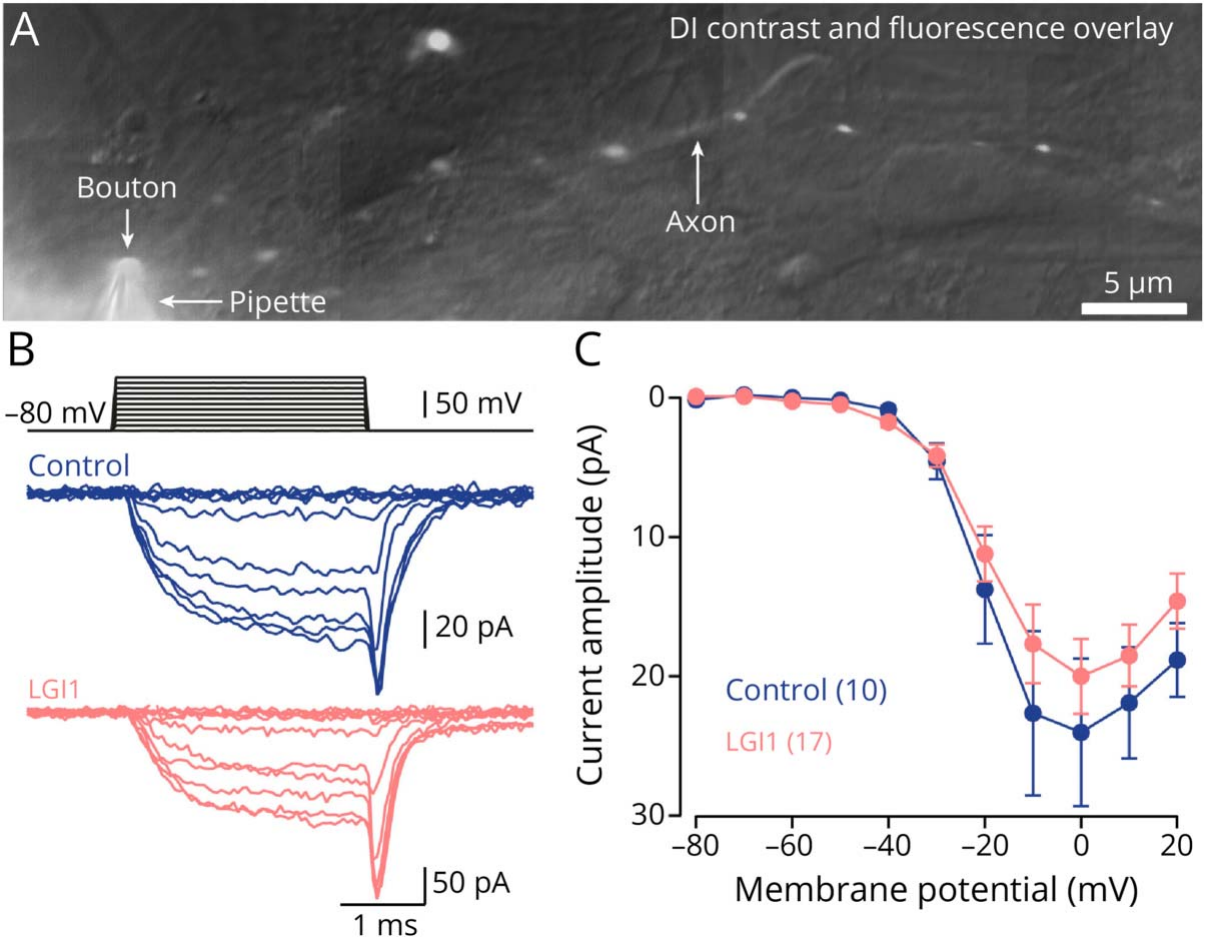
LGI1 Autoantibodies Do Not Affect Presynaptic Calcium Channel Gating (A) Overlay of a difference-interference (DI) contrast and fluorescence image (200 µM Atto 488 contained in recording pipette) of a small bouton whole-cell recording in a primary hippocampal culture. (B) Example traces of pharmacologically isolated calcium currents evoked by 3 ms depolarizations to different voltages in control antibody-treated (blue) and LGI1 autoantibody-treated boutons (rose). (C) Calcium current amplitudes during the final 1 ms of the step depolarizations in (B). Numbers in brackets reflect recordings from individual presynaptic boutons. Dots reflect mean ± SEM current amplitudes.

The LGI1-receptor ADAM22 has been shown to interact with various Ca_v_ channel beta-subunits,^18^ which in turn affect calcium current gating.^40^ We therefore determined the effect of LGI1 autoantibodies on calcium channel gating by directly measuring pharmacologically isolated presynaptic calcium currents in whole-cell voltage-clamp recordings from boutons in hippocampal cultures (Figure 3, A and B). Calcium currents on 3 ms depolarization were similar in amplitudes for control and LGI1 autoantibody-treated boutons (Figure 3C; median [IQR] current amplitude at 0 mV: 18.1 [10.5–28.4] pA and 18.3 [10.5–25.3] pA, n = 10 and 17, for control and LGI1, respectively, *p* = 0.66; 2-way ANOVA for overall effect: *p* = 0.079). In addition, the time course of current activation was not affected by LGI1 autoantibodies (eFigure 4, *p* = 0.86). Similarly, amplitude and time course of calcium current inactivation were unchanged following LGI1 autoantibody treatment (eFigure 4). Unaltered presynaptic calcium currents indicate that LGI1 autoantibodies did not affect calcium channel gating and thus cannot explain the increased release probability by LGI1 autoantibodies.

### Nanoscale Localization of Presynaptic K_v_1.1 and K_v_1.2 Channels in Hippocampal Synapses

**Figure 4:**
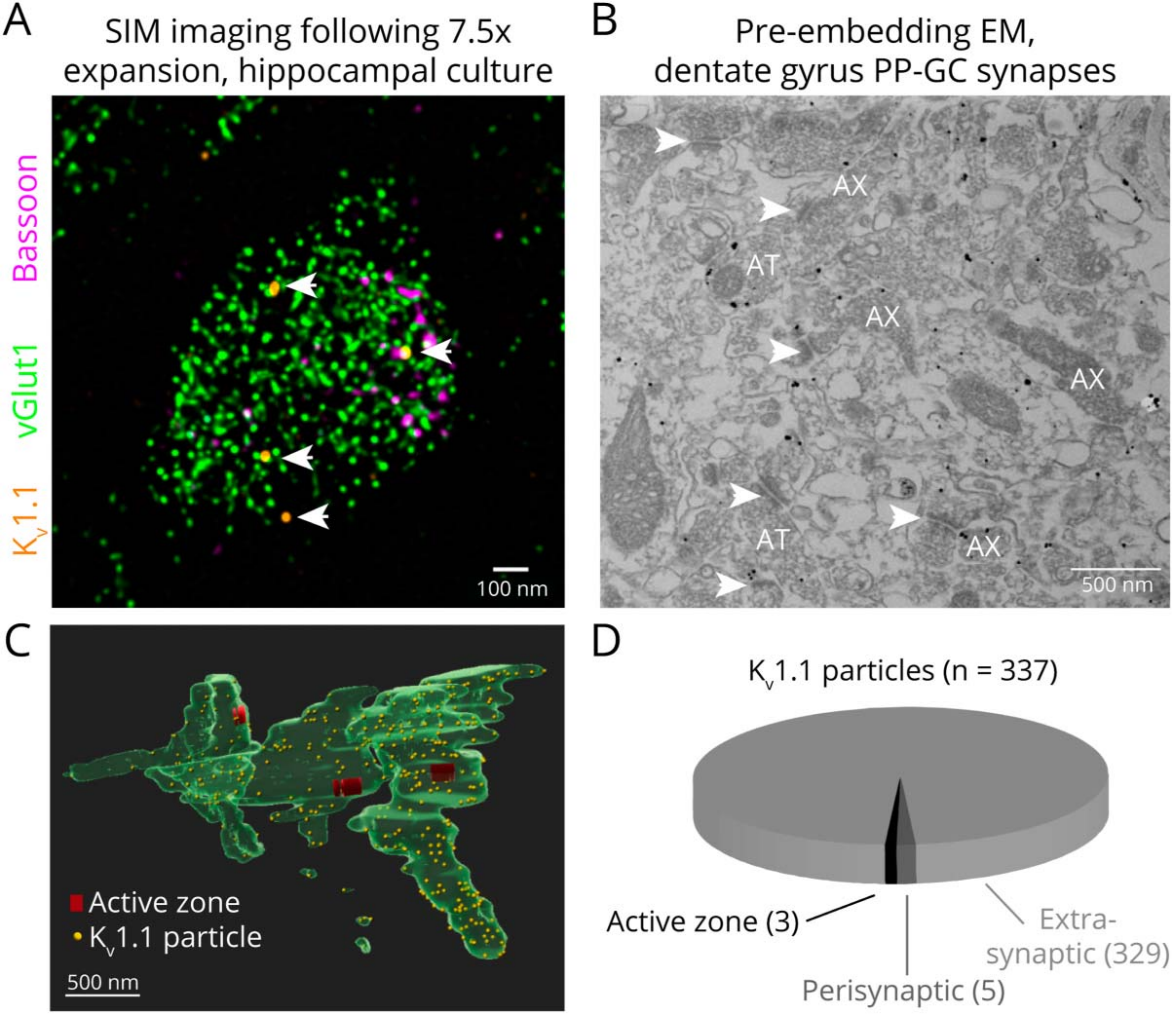
Nanoscale Localization of Presynaptic Kv1.1 Channels in Hippocampal Synapses (A) Example structured illumination microscopy (SIM) image of a ∼7.5-fold expanded presynapse in primary hippocampal cultures triple-stained for vGlut1 (green), Bassoon (magenta), and K_v_1.1 (orange). Arrows indicate K_v_1.1 channels within and outside of the Bassoon-labeled active zone. (B) Example pre-embedding electron microscopic image of a dentate gyrus molecular layer section immunogold-labelled for K_v_1.1 (AX = axon, AT = axon terminal; arrowheads depict excitatory synapses with postsynaptic densities). (C) 3D reconstruction of axons and their terminals harboring multiple active zones (red) and K_v_1.1 particles (yellow spheres). (D) Quantification of K_v_1.1 particle localization within active zones (black), the perisynaptic space (dark gray; ≤60 nm from the active zone edge), and the extrasynaptic space (light gray; >60 nm from the active zone edge). Numbers in brackets indicate total particle counts or counts within respective localizations.

LGI1 interacts through ADAM22-receptors with K_v_1 potassium channels,^17,18^ which are reduced in animals treated with LGI1 autoantibodies.^27,30^ We therefore hypothesized that the release probability is increased because of the loss of presynaptic K_v_1 channels. We first tested whether K_v_1.1 and K_v_1.2 channel subtypes, which have been linked to LGI1 functionally and in biochemical assays,^11,17,18^ are localized presynaptically in cultured hippocampal neurons. Using SIM of neurons co-stained for Bassoon, we found that K_v_1.1 and K_v_1.2 channels were localized at presynaptic active zones in cultured hippocampal neurons (eFigure 5; Mander colocalization coefficients for K_v_1.1 and Bassoon = 0.28 ± 0.11 (mean ± SD), n = 137 synapses; for K_v_1.1 and Bassoon 0.62 ± 0.13, n = 195 synapses). To test potential limitations of the spatial resolution, we used postgelation expansion and immunolabeling in combination with SIM (Ex-SIM).^33^ Samples expanded ∼7.5-fold, thus enabling a spatial resolution of ∼20 nm by multicolor SIM. Again, both K_v_1.1 and K_v_1.2 channels localized at vGlut1-positive presynaptic nerve terminals of cultured hippocampal neurons (Figure 4A and eFigure 5). Furthermore, both K_v_1.1 and K_v_1.2 were found inside and outside of the Bassoon-labeled active zone in cultured hippocampal neurons (eFigure 5). To corroborate the presence of K_v_1 channels at hippocampal presynapses in brain tissue, we studied hippocampal dentate gyrus perforant path-granule cell synapses in perfusion-fixed hippocampal tissues. At these synapses, it was previously shown that LGI1 antibodies also increase the release probability.^27^ Using pre-embedding electron microscopy, we localized K_v_1.1 channels (Figure 4B) and K_v_1.2 channels (eFigure 5) at the perforant path-granule cell synapses. A three-dimensional reconstruction of perforant path axon terminals and the adjacent axons indicated a homogeneous K_v_1.1 channel distribution (Figure 4C), consistent with <5% of both channel subtypes localized at or close to the small surface area building the presynaptic active zone (*active zone* and *perisynaptic*; Figure 4D and eFigure 5). However, the large majority of the K_v_1.1 and K_v_1.2 channels were found outside of the active zone. Thus, complementary high-resolution light and electron microscopic techniques confirm the localization of both K_v_1.1 and K_v_1.2 channels at hippocampal presynapses and indicate a rather homogeneous distribution.

### LGI1 Autoantibodies Reduce Presynaptic K_v_1.1 and K_v_1.2 Channels

After we found presynaptic localization of K_v_1.1 and K_v_1.2 channels, we tested whether their localization was affected by LGI1 autoantibodies. LGI1 autoantibodies were previously shown to reduce general synaptic K_v_1.1 channels using confocal imaging^27^ or western blots.^30^ We first investigated the co-localization of bound pathogenic LGI1 autoantibodies and K_v_ channels using confocal and STED microscopy (eFigure 6). LGI1 autoantibody localization showed a punctate pattern. Co-localization of LGI1 puncta with K_v_1.1 and K_v_1.2 at the soma and dendrites was weak, but the majority of LGI1 puncta had at least a weak K_v_ signal, while many K_v_ puncta had no LGI1 signal. Furthermore, we found that both K_v_1.1 and K_v_1.2 showed a strong signal at the axon initial segment (AIS), which was identified by the presence of Ankyrin G (AnkG) and a coincident lack of Microtubule-associated protein 2 (MAP2; eFigure 7). The K_v_1.1 and K_v_1.2 staining in the AIS showed 190 nm spaced bands (eFigure 7) as described for other proteins at the AIS^41^; however, LGI1 and K_v_ colocalization at the AIS was weak. These results are not surprising because LGI1 might be secreted (but see ref. 42) and the interaction of LGI1 with K_v_1 channels, possibly through ADAM-family proteins, is still not well understood.^14^ Our data thus argue against a fixed stoichiometric interaction of LGI1 with K_v_ channels.

**Figure 5:**
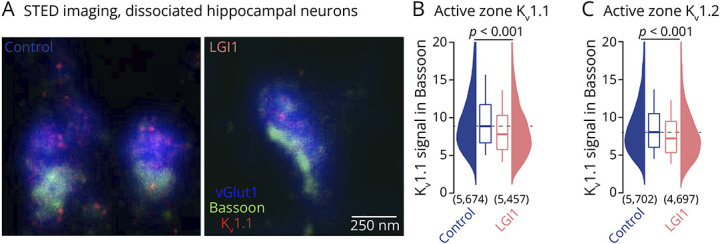
LGI1 Autoantibodies Reduce Presynaptic Kv1.1 and Kv1.2 Channels (A) STED fluorescence images of primary hippocampal presynapses stained for vGlut1 (blue), Bassoon (green), and K_v_1.1 (red) following treatment with control antibodies (left) or LGI1 autoantibodies (right). (B) Active zone K_v_1.1 fluorescence intensity (within Bassoon) for control antibody (blue) and LGI1 autoantibody (rose) treatment. (C) Active zone K_v_1.2 fluorescence intensity (within Bassoon) for control antibody and LGI1 autoantibody treatment (color code as in B). Box plots provide median and cover percentile 25–75, whiskers reflect percentiles 10–90. Broken lines indicate the respective control condition median intensity. Numbers in brackets provide the number of analyzed presynapses. *p* Values were calculated using the Mann-Whitney *U* test.

To analyze the effect of treatment with pathogenic LGI1 antibodies on K_v_1.1 and K_v_1.2 channels, we performed STED imaging because of its higher resolution compared with confocal microscopy and the higher throughput compared with the Ex-SIM technique. We investigated K_v_1.1 and K_v_1.2 channels at excitatory presynapses in cultured hippocampal neurons (Figure 5A). Because of the variability in K_v_1 signal intensity between synapses, we repeated antibody application, immunostaining, image acquisition, and image analyses in 10 cultures independently generated from 10 different animals. LGI1 autoantibodies reduced K_v_1.1 (Figure 5B) and K_v_1.2 signals (Figure 5C) at excitatory active zones by 10%–15% (median change for K_v_1.1 within Bassoon −12.3%, K_v_1.2 within Bassoon −10.2%, each *p* < 0.001 and n = ∼5000 synapses). Similarly, K_v_1.1 and K_v_1.2 signals were reduced within the vGlut1-labeled presynaptic boundary (eFigure 8). Even when we analyzed each culture separately (which might represent an over-critical definition of the biological replicate), the LGI1 autoantibodies showed trends of reduction or statistically significant reduction of both, K_v_1.1 and K_v_1.2, within vGlut1 and Bassoon (eFigure 8). Consistent with a homogeneous distribution of K_v_1.1 and K_v_1.2 in electron microscopy, we also observed a reduction in the density of presynaptic K_v_1.1 and K_v_1.2 outside of the active zone (i.e., inside the vGlut1 but outside of the Bassoon mask; data not shown). Thus, presynaptic K_v_1.1 and K_v_1.2 channels are both homogeneously reduced within the presynaptic terminal on treatment with LGI1 autoantibodies.

### LGI1 Autoantibodies Lead to Increased Presynaptic Action Potential Broadening

**Figure 6:**
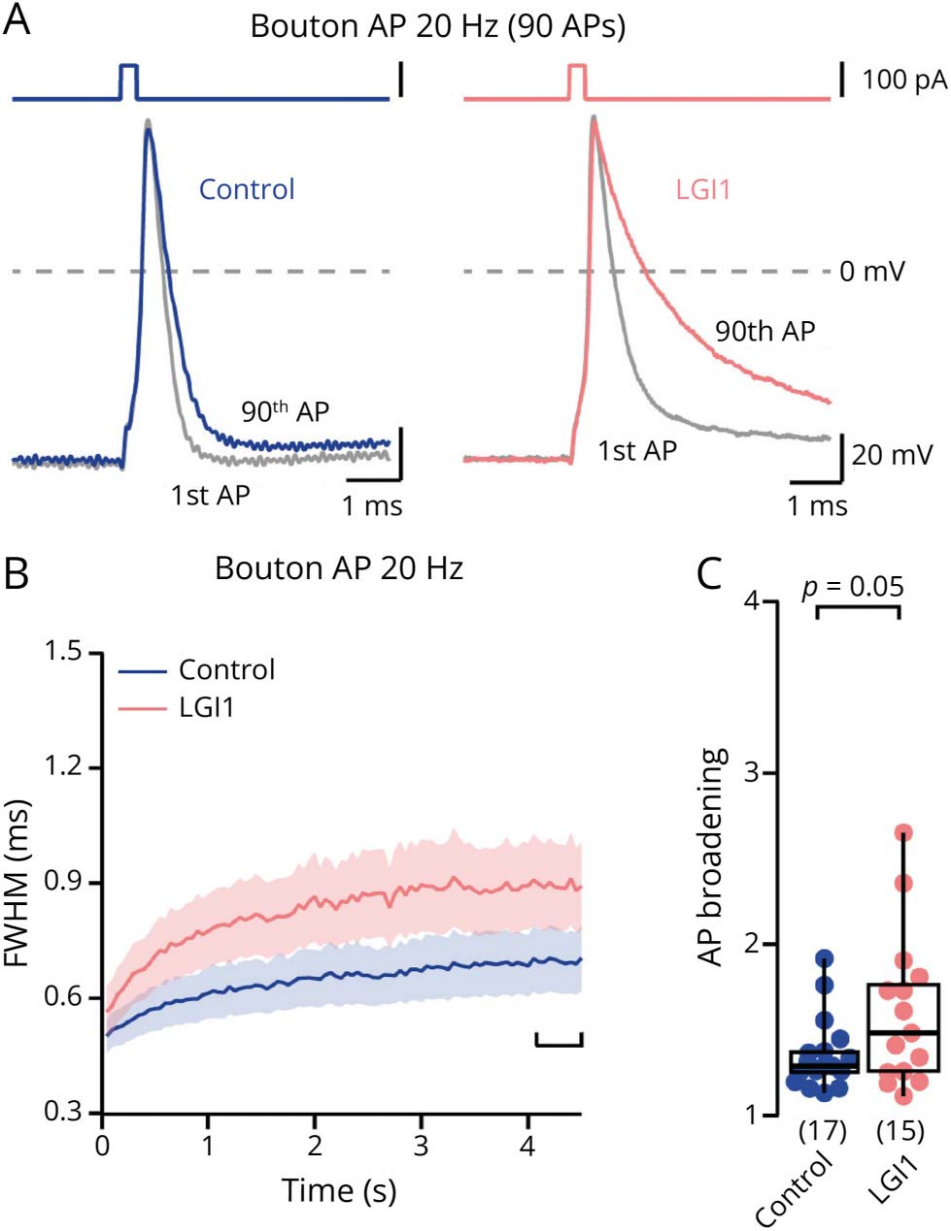
LGI1 Autoantibodies Lead to Increased Presynaptic Action Potential Broadening (A) Overlay of first and last action potential of an action potential train (90 action potentials evoked at 20 Hz) following treatment with control antibodies (blue) or LGI1 autoantibodies (rose). (B) Action potential broadening (mean ± SEM FWHM, normalized to the FWHM of the first action train potential) during 20 Hz train stimulation in control and LGI1 autoantibody-treated presynaptic boutons (color code as in A). (C) Magnitude of action potential broadening (mean normalized FWHM of the 10 last train action potentials) during 20 Hz train stimulation in control and LGI1 autoantibody-treated presynaptic boutons (color code as in A). Box plots provide median and cover percentile 25–75, whiskers reflect percentiles 10–90. Numbers in brackets provide number of recorded presynaptic boutons. The *p* values were calculated using the Mann-Whitney *U* test.

K_v_1 channels control presynaptic action potential duration (e.g., see ref. 42 and references therein). To determine the functional relevance of presynaptic K_v_1 channel loss, we performed direct current-clamp recordings from boutons in hippocampal cultures^43,44^ following autoantibody treatment. Action potentials evoked by current injections had large amplitudes and short half-durations (quantified as full-width recorded at half-maximal amplitude, FWHM), similar to previous findings at boutons of neocortical cultures.^43^ Changes in action potential shape were tested by evoking trains of 90 action potentials at 20 or 50 Hz (Figure 6A). Action potential broadening was pronounced following LGI1 autoantibody treatment during 20 Hz train stimulation (Figure 6, B and C; 20 Hz: median [IQR] broadening of the last 10 action potentials: 29.1 [25.0–37.7] % and 48.4 [25.6–77.1] %, n = 17 and 15 for control and LGI1, respectively, *p* = 0.05) and 50 Hz train stimulation (*p* = 0.03; eFigure 9). Owing to the large bouton-to-bouton variability, the absolute duration of the last 10 action potentials only showed a trend toward an increase duration (*p* = 0.23 and *p* = 0.16 for 20 and 50 Hz, respectively; data not shown). In contrast to the duration of action potentials, the amplitudes of presynaptic action potentials were not affected by LGI1 autoantibodies (eFigure 9; change in median amplitude of last 10 action potentials <3% at 20 Hz and <10% at 50 Hz, both *p* > 0.05). Besides changes in action potential broadening, treatment with LGI1 autoantibodies also increased bouton excitability, leading to aberrant action potential firing during current injections (eFigure 9; repetitive action potentials on prolonged current injections in 1/15 and 5/13 boutons for control and LGI1, respectively, *p* = 0.04). Similar to presynaptic action potentials, somatic action potentials were broadened and showed increased activity-induced broadening following treatment with LGI1 autoantibodies (eFigure 9). It is well established that broader presynaptic action potentials lead to more calcium influx and higher release probability.^45-47^ Thus, these data indicate that by reducing K_v_1 channels, LGI1 autoantibodies enhance somatic and presynaptic action potential broadening and thus synaptic release probability.

### Autoantibodies Targeting the EPTP Domain but Not the LRR Domain of LGI1 Cause Action Potential Broadening

**Figure 7:**
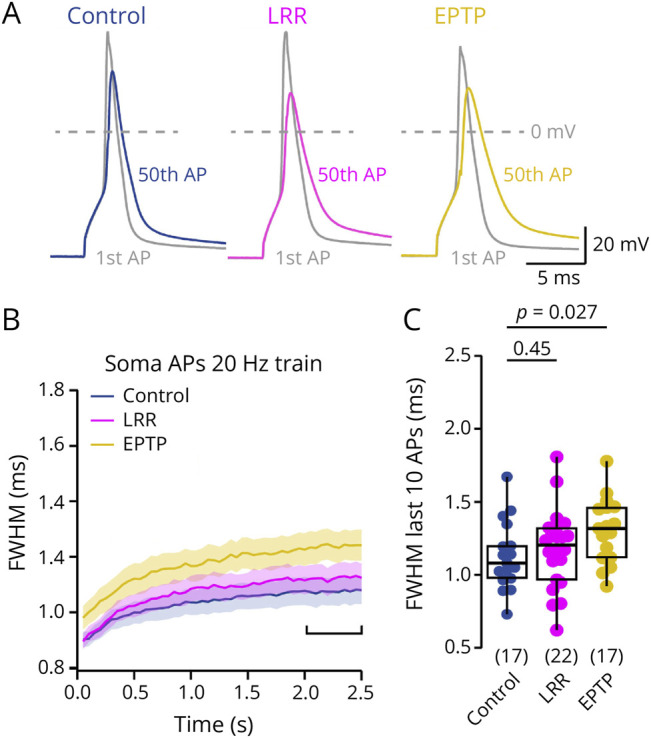
Autoantibodies Targeting the EPTP Domain but Not the LRR Domain of LGI1 Cause Action Potential Broadening (A) Overlay of first and last action potentials of a somatic action potential train (50 action potentials evoked at 20 Hz) following treatment with control antibodies (blue), LRR autoantibodies (magenta), or EPTP autoantibodies (yellow). (B) Time course of somatic action potential broadening (mean ± SEM FWHM) during 20 Hz trains following treatment with control antibodies, LRR autoantibodies, or EPTP autoantibodies (color code as in A). (C) Magnitude of action potential broadening (mean FWHM of the 10 last train action potentials) during 20 Hz train stimulation following treatment with control antibodies, LRR autoantibodies, or EPTP autoantibodies (color code as in A). Box plots provide median and cover percentile 25–75, whiskers reflect percentiles 10–90. Numbers in brackets provide number of recorded somata. The *p* values were calculated using the Kruskal-Wallis tests followed by Dunn multiple comparison tests.

To address which of the 2 main LGI1 domains is involved in the antibody-mediated action potential broadening, we again recorded somatic action potentials, this time however following treatment with patient-derived monoclonal autoantibodies specifically targeting only either the EPTP or the LRR domain (see supplementary material, eMethods, for details on the antibodies).^31^ We again first tested the colocalization of the pathogenic monoclonal LGI1 autoantibodies with K_v_1.1 and K_v_1.2 channels (eFigure 6). Similar to the polyclonal antibodies, there was little colocalization. In addition, there was little overall binding of the anti-EPTP antibodies. This is consistent with the original description of these antibodies in which the EPTP antibodies inhibited the docking of LGI1 to ADAM22/23 and induced pronounced memory defects, but the surface binding of EPTP antibodies was much lower compared with the LRR antibodies.^30^ Compared with cells treated with control isotype matching monoclonal antibodies, LRR autoantibodies did not affect action potential broadening during 20 Hz trains (Figure 7, A–C; median [IQR] FWHM of last 10 train action potentials at 20 Hz: 1.08 [0.98–1.20] ms and 1.20 [0.99–1.31] ms, n = 17 and 22 for control and LLR, respectively, *p* = 0.45) and 50 Hz trains (eFigure 10). By contrast, treatment with EPTP autoantibodies led to enhanced action potential broadening during 20 and 50 Hz trains (Figure 7, A–C and eFigure 10; median [IQR] FWHM of last 10 train action potentials at 20 Hz: 1.32 [1.12–1.46] ms for EPTP, n = 17, *p* = 0.027). The broadening of somatic action potentials following EPTP autoantibody treatment was similar in magnitude to the broadening induced by polyclonal LGI1 autoantibodies (cf. Figure 7, B and C and eFigures 9 and 10), suggesting that antibody binding to the EPTP domain underlies action potential broadening.

## Discussion

Our results have important implication for understanding the pathophysiology of LGI1 autoimmune encephalitis and the physiologic functions of LGI1. In particular, our study demonstrates that (1) LGI1 autoantibodies broaden presynaptic action potentials, which explains the observed increase in release probability. (2) We did not find relevant changes in the density nor the gating of calcium channels on LGI1 autoantibody treatment. (3) The homogeneous presynaptic distribution and reduction of K_v_1.1 and K_v_1.2 channels on LGI1 autoantibody treatment indicate that LGI1 can act outside of the release site in addition to its transsynaptic function. (4) Experiments with domain-specific patient-derived monoclonal autoantibodies indicate that action potential broadening is mediated by autoantibodies targeting the EPTP domain but not by antibodies targeting the LRR domain. Thus, our study provides a mechanistic framework explaining the neuronal hyperactivity of patients with LGI1 antibody encephalitis.

It is controversial whether LGI1 autoantibodies affect synaptic transmission presynaptically, postsynaptically, or both, presynaptically and postsynaptically. We found that LGI1 autoantibodies decreased paired-pulse ratios and increased synaptic depression arguing for a presynaptic effect of LGI1 autoantibodies.^48^ Our results are consistent with previous studies reporting that LGI1 autoantibodies increase synaptic strength and decrease paired-pulse ratio at hippocampal perforant path-granule cell synapses and reduce synaptic failures in CA1 neurons,^27^ with a similar trend toward higher mEPSC frequency observed in CA3 neurons.^29^ Synapses onto hippocampal CA1 and CA3 neurons were not affected in strength or paired-pulse ratio by LGI1 autoantibodies,^25,27,28,30^ which might be due to lower abundance of the LGI1 protein at these synapses.^17^ The increased release probability on LGI1 antagonism provides an explanation for the hyperactivity in both LGI1 autoantibody-treated neurons^25,28^ and neurons of LGI1 knock-out mice.^11,20,21,49^ Furthermore, the increased release probability might also serve as a basis for the epileptic seizures of patients suffering from LGI1 antibody encephalitis.^50^ The faciobrachial dystonic seizures respond intriguingly fast to immunotherapy, whereas antiseizure medication is often ineffective.^4,51^ Our data suggest that the ineffectiveness of antiseizure medication could be due to the direct, antibody-induced increase in presynaptic function. More studies are needed to better understand the underlying cause of seizures in anti-LGI1 encephalitis to develop effective causative and symptomatic treatment.

LGI1 and ADAM receptor proteins have previously been shown to affect K_v_1 channel gating^17^ and expression,^16,52^ and LGI1 autoantibodies immunoprecipitate with K_v_ channels.^3,15^ We found that both K_v_1.1 and K_v_1.2 subunits were localized presynaptically, consistent with previous results on K_v_1 channel localization.^53^ LGI1 autoantibodies reduced presynaptic K_v_1.1 and K_v_1.2 channels, in agreement with reduced hippocampal K_v_1.1 fluorescence^27^ and K_v_1 protein levels^30^ following autoantibody treatment. Direct bouton patch-clamp recordings revealed enhanced action potential broadening during train stimulation, a well-known consequence of reduced K_v_1 conductance on activity-dependent K_v_1 channel inactivation^45,46^ or pharmacologic K_v_1 channel block (e.g., see ref. 42 and references therein). Therefore, our presynaptic structural-functional analysis provides direct support for the following mechanistic steps: (1) LGI1 autoantibodies interfere with LGI1's endogenous function of increasing the presynaptic potassium channels density. (2) The reduction of presynaptic potassium channels prevents efficient repolarization of the presynaptic action potential. (3) The resulting longer presynaptic action potential increases release probability.

Consistent with increased release probability on antibody application, knock-out of LGI1 in mice increased transmission at hippocampal CA3-CA3 synapses^16^ and in CA1 neurons.^21,54^ Furthermore, overexpression of LGI1 decreased synaptic strength at perforant-path granule cell synapses.^19^ The synaptic strengthening on LGI1 knock-out is probably mediated presynaptically by an increased release probability because LGI1 knock-out postsynaptically either decreased AMPAR clustering and quantal size^11,22,24,26^ or did not affect quantal size.^19,21^ However, some synapses show no presynaptic effect on LGI1 knock-out or LGI1 application. For example, in CA1 neurons, PPR was mostly unaffected by LGI1 application^11^ or LGI1 knock-out.^11,22,24,54^ These differences in the presynaptic effect of LGI1 knock-out on synaptic transmission may relate to the differential expression of LGI1, with highest expression in the hippocampal outer and middle molecular layers of the denate gyrus (perforant path-granule cell synapses).^17^ Furthermore, LGI1-overexpression shortened presynaptic action potentials in primary hippocampal cultures, leading to lower action potential-evoked calcium entry and hence glutamate release.^42^ Therefore, LGI1 autoantibodies induce effects that are reminiscent of those observed in LGI1 knock-out mice and thus support the mechanistic model that LGI1 increases the presynaptic potassium channel density, shortens the presynaptic action potential duration, lowers the release probability, and thereby dampens neuronal activity.

We found that K_v_1 channels are homogeneously distributed across the axon and bouton and only a minority of potassium channels was located at the presynaptic release site (Figure 4 and eFigure 5). Furthermore, LGI1 autoantibodies decreased the K_v_1 density within and outside of the Bassoon-labeled release sites, indicating a homogeneous reduction throughout the bouton (Figure 5 and eFigure 8). Our data therefore argue that LGI1, in addition to its transsynaptic alignment, controls potassium channels also outside of the release site. Indeed, it was recently shown that LGI1 autoantibodies also alter the K_v_1 cluster distribution at the axon initial segment.^55,56^ The autoantibody-induced increase in neuronal excitability was mediated by antibodies specifically targeting the LRR domain.^29,30,55,56^ Consistently, structural analyses indicate that LGI1 can form protein complexes in a cis-configuration serving as an extracellular scaffold instead of a transsynaptic hub.^14,57^ It remains to be determined if the density of presynaptic K_v_1 channel outside of the release site is controlled by LGI1 proteins in the cis-configuration.

The analyses of calcium channels were motivated by the increase in synaptic release probability following LGI1 autoantibody treatment, a phenomenon typically observed on changes in calcium channel density or function. Furthermore, proteome data previously indicated an interaction of the LGI1-receptor ADAM22 with pore-forming calcium channel alpha-subunits and their beta-subunits,^18,37^ which control calcium channel surface expression and kinetics.^40,58^ We used STED and EM imaging of Ca_v_2.1 calcium channels and direct electrophysiologic recordings of presynaptic calcium current density and gating kinetics. Yet, we found neither presynaptic Ca_v_2.1 channel abundance nor calcium current amplitude and channel gating kinetics were strongly affected by LGI1 autoantibodies (if anything, there was a reduction in the channel density). Therefore, potential effects of LGI1 on presynaptic calcium channels do not contribute to the increased release probability induced by LGI1 autoantibodies.

Previously, patient-derived monoclonal autoantibodies were used to specifically target the EPTP or the LRR domain of LGI1.^30^ Although EPTP-targeting autoantibodies led to enhanced broadening during train stimulation in our recordings, LRR autoantibodies did not affect action potential broadening. The EPTP domain of LGI1 has been shown to mediate binding to ADAM22, and EPTP-targeting autoantibodies hence prevented binding of LGI1 to ADAM22.^12,26,29,30^ Both EPTP and LRR autoantibodies reduced K_v_1.1 protein levels in hippocampus-enriched solubilized brain lysates, but the effect seemed stronger with EPTP—compared with LRR autoantibodies.^30^ By contrast, some studies observed an increased neuronal excitability only with LRR but not with EPTP autoantibodies^55,56^ or a stronger effect on excitability with LRR compared with EPTP antibodies.^29^ LRR-targeting antibodies were previously shown to interfere with multimerization and cause internalization of the LGI1-ADAM22 complex.^12,29,30^ A differential effect of autoantibodies targeting EPTP and LRR is conceivable because of the complex interplay of various types of potassium channels in controlling excitability and action potential repolarization.^59^ However, more studies are needed to understand the differential effect of the subunit-specific autoantibodies on excitability and action potential repolarization. Furthermore, although we tested 2 monoclonal antibodies for each LGI1 functional domain, our data cannot rule out that LRR autoantibodies with different binding epitopes other than those tested here are able to affect presynaptic K_v_1 function. Indeed, there might be differences with the subclones used in previous studies.^29,56^ However, we use the exact same set of antibodies as in Sell et al.,^55^ who also found stronger effects with LLR antibodies on excitability as previous studies.^29,56^ It is difficult to rule out that due to technical reasons, the antibodies change their potency. However, our data (eFigure 6) argue against the possibility that the absence of action potential broadening with LRR antibodies is due to a lost binding ability of the antibodies. Taken together, the data thus suggest differences in the regulation of excitability and action potential duration by the 2 domains of LGI1, which could be reflected in differential symptoms associated with mutations in these domains, such as auditory features that occur less frequently in congenital epilepsy caused by EPTP truncation compared with LRR truncation.^60^

## Appendix Authors

**Table:**

Name	Location	Contribution
Andreas Ritzau-Jost, MD	Carl-Ludwig-Institute of Physiology, Faculty of Medicine, Leipzig University, Germany	Drafting/revision of the manuscript for content, including medical writing for content; major role in the acquisition of data; study concept or design; analysis or interpretation of data
Felix Gsell	Carl-Ludwig-Institute of Physiology, Faculty of Medicine, Leipzig University, Germany	Major role in the acquisition of data; analysis or interpretation of data
Josefine Sell, PhD	Section Translational Neuroimmunology, Department of Neurology, Jena University Hospital, Germany	Drafting/revision of the manuscript for content, including medical writing for content; major role in the acquisition of data; analysis or interpretation of data
Stefan Sachs	Department of Biotechnology and Biophysics, University of Würzburg, Biocenter, Germany	Major role in the acquisition of data; analysis or interpretation of data
Jacqueline Montanaro, PhD	Institute of Science and Technology Austria (ISTA), Klosterneuburg, Austria	Major role in the acquisition of data; analysis or interpretation of data
Toni Kirmann	Carl-Ludwig-Institute of Physiology, Faculty of Medicine, Leipzig University, Germany	Major role in the acquisition of data; analysis or interpretation of data
Sebastian Maaß, PhD	Carl-Ludwig-Institute of Physiology, Faculty of Medicine, Leipzig University, Germany	Major role in the acquisition of data; analysis or interpretation of data
Sarosh R. Irani, DPhil	Oxford Autoimmune Neurology Group, Nuffield Department of Clinical Neurosciences, University of Oxford; Department of Neurology, John Radcliffe Hospital, Oxford University Hospitals, United Kingdom; Departments of Neurology and Neurosciences, Mayo Clinic Jacksonville, FL	Drafting/revision of the manuscript for content, including medical writing for content; study concept or design
Christian Werner, PhD	Department of Biotechnology and Biophysics, University of Würzburg, Biocenter, Germany	Drafting/revision of the manuscript for content, including medical writing for content; analysis or interpretation of data
Christian Geis, MD	Section Translational Neuroimmunology, Department of Neurology, Jena University Hospital, Germany	Drafting/revision of the manuscript for content, including medical writing for content; study concept or design; analysis or interpretation of data
Markus Sauer, PhD	Department of Biotechnology and Biophysics, University of Würzburg, Biocenter, Germany	Drafting/revision of the manuscript for content, including medical writing for content; analysis or interpretation of data
Ryuichi Shigemoto, MD, PhD	Institute of Science and Technology Austria (ISTA), Klosterneuburg, Austria	Drafting/revision of the manuscript for content, including medical writing for content; study concept or design; analysis or interpretation of data
Stefan Hallermann, MD	Carl-Ludwig-Institute of Physiology, Faculty of Medicine, Leipzig University, Germany	Drafting/revision of the manuscript for content, including medical writing for content; major role in the acquisition of data; study concept or design; analysis or interpretation of data

## Glossary

AIS: axon initial segment
EPSCs: excitatory postsynaptic currents
LGI1: leucine-rich glioma inactivated 1
LRR: leucine-rich repeat
PPR: paired-pulse ratio
SIM: structured illumination microscopy
STED: stimulated emission depletion
